# Single Nucleotide Polymorphisms in *COX2* Is Associated with Persistent Primary Tooth and Delayed Permanent Tooth Eruption

**DOI:** 10.3390/ijerph191610047

**Published:** 2022-08-15

**Authors:** Erika Calvano Küchler, Suelyn Danielle Henklein, Peter Proff, César Penazzo Lepri, Camila Paiva Perin, Eva Paddenberg, Liliane Roskamp, Flares Baratto-Filho, Maria Angélica Hueb de Menezes-Oliveira, Christian Kirschneck

**Affiliations:** 1 Department of Orthodontics, University of Regensburg, Franz-Josef-Strauss-Allee 11, 93053 Regensburg, Germany; 2 School of Dentistry, Tuiuti University of Paraná, Rua Sydnei Antonio Rangel Santos, 238-Santo Inacio, Curitiba 82010-330, PR, Brazil; 3 Department of Biomaterials, University of Uberaba-UNIUBE, Uberaba 38010-200, MG, Brazil; 4 School of Dentistry, Univille University, Rua Paulo Malschitzki, 10, Zona Industrial Norte, Joinville 89219-710, SC, Brazil

**Keywords:** children, primary tooth, genes

## Abstract

Persistent primary tooth (PPT) is a prevalent clinical condition that occurs when a primary tooth is over-retained beyond the established period of its normal exfoliation time, remaining in the oral cavity. Many factors could be involved in the risk of PPT; therefore, the aim of this study was to evaluate if single nucleotide polymorphisms (SNPs) in the *COX2* gene are associated with PPT. Children undergoing orthodontic treatment were screened. Orthopantomographs were assessed to evaluate PPT according to the Nolla stage of its permanent successor. The primary tooth was considered retained when its successor permanent tooth was in Nolla stage 8 and below the alveolar crypt, Nolla stage 9, or Nolla stage 10. A saliva sample from each child was collected and used for DNA extraction. A real-time PCR of two SNPs, rs689466 (−1195 G/A) and rs5275 (+665 T/C), was performed. A chi-square test was used to compare the allele and genotype distribution. Haplotype analysis was also performed. A total of 100 children were included in the study. Fifty-one had at least one PPT, while 49 children were classified as a control. The number of teeth persistent in the oral cavity ranged from 1 to 8. The genotype distribution was associated with PPT in the co-dominant model (*p* = 0.006) for SNP rs5275. The individuals that carry two T alleles (TT) compared with the individuals that carry at least one C allele (C + TC) had an almost three times higher chance of presenting with PPT (*p* = 0.012; OR = 2.99, CI95% 1.28 to 6.95–recessive model). The haplotype C-A for the SNPs rs5275 and rs689466, respectively, was significantly associated (*p* = 0.042). In conclusion, single nucleotide polymorphisms in the gene encoding for *COX2* are associated with persistent primary tooth and may delay permanent tooth eruption.

## 1. Introduction

Permanent and primary tooth eruptions are both fundamental developmental and physiologic processes that occur during a child’s growth and development. Permanent and primary tooth eruption and root elongation during dental development require the resorption of surrounding alveolar bone to occur. In some permanent teeth (incisors, canines, and premolars) eruption, the root resorption of the corresponding primary tooth also occurs [1]. Root resorption of the primary tooth is a physiological process in the life span of a primary tooth and results in its exfoliation leading to the eruption of its successor tooth [2]. Additionally, bone remodeling during the establishment of the eruption pathway involves bone resorption on the occlusal surface and bone formation in the basal region of the developing root [1]. The root resorption of the primary tooth is a process regulated similarly to the bone remodeling process at the molecular level, sharing several common genes [2].

Persistent primary tooth (PPT) is a prevalent clinical condition in children and teenagers that occurs when a primary tooth is over-retained beyond the established period of its normal exfoliation time, remaining in the child’s oral cavity and commonly leading to a delay in the permanent successor tooth [3,4]. Many encoding genes involved in the bone remodeling process and primary root resorption process are candidate genes for PPT, including Cyclooxygenase-2 (*COX2*), which is an important enzyme mediating prostaglandin synthesis. COX2 is encoded by the Prostaglandin-Endoperoxide Synthase 2 *(PTGS2*) gene, which can also be called the *COX2* gene. *COX2* is well-known to play a key role in the alveolar bone resorption process [5]. In a study evaluating the periodontal ligament of human permanent teeth and primary teeth undergoing root resorption, Howship lacunae were found only in primary teeth and were associated with the presence of TRAP-positive cells and an increase in COX2 expression [6].

The *COX2* gene is located on chromosome 1q25.2–q25.3 and contains 10 exons and encodes a 604-amino-acid protein [5]. Single Nucleotide Polymorphisms (SNPs) are a type of genetic variant involving changes of a single base pair in the genome. This is the most common type of genetic variation in humans, and these variants could explain the differences in an individual’s predisposition to present with complex traits [7] such as PPT. Although the etiology of PPT has been poorly investigated, the studies showing the molecular background of tooth eruption clearly support the assumption that SNPs in key genes could be involved in PPT. In this study, we hypothesize that common SNPs in the *COX2* signaling pathway may act as potential predictors of PPT risk. Therefore, we evaluated two well-known SNPs in the *COX2* encoding gene in a group of children with PPT associated with delayed permanent tooth eruption.

## 2. Materials and Methods

### 2.1. Ethics and Sample Description

This study was previously approved by the Ethics Committee of the University Hospital Regensburg, Germany (ID 19-1549-101). All of the included children and teenagers and/or their legal guardians were informed about all aspects of the research. The patients were included in the study after their legal guardians signed the informed consent form and the literate children signed an assent form. All of the patients were treated in accordance with the Declaration of Helsinki. The STrengthening the REporting of Genetic Association Studies (STREGA) statement checklist was followed to design this study protocol and to report the results presented here [8].

Biologically-unrelated children undergoing orthodontic treatment at the Orthodontic Department at the University of Regensburg and two collaborating private orthodontic practices in the area (Regensburg, Germany), between 2019 and 2021, were screened, selected, and included in the study. Only children aged between 8 and 14 years with a Central-European ancestry (at maximum one grandparent not from Central Europe) were selected. Patients with tooth agenesis (excluding third molar agenesis), facial trauma, cleft lip and/or cleft palate, underlying syndromes, and/or systemic diseases were excluded from the study. Only one child per family was included to avoid bias.

### 2.2. Panoramic Radiographs Evaluation and Characterization of PPT

Panoramic radiographs (orthopantomographs) of each child were assessed by a single examiner, who is a senior pediatric dentist. First, a calibration was performed using 10 panoramic radiographs, which were evaluated twice, two weeks apart. The intra-examiner calibration was calculated, and the Kappa value was 0.98 for Nolla’s stage.

The primary teeth associated with the following conditions were excluded from the analysis, and they were not considered to be PPT: Ectopic tooth eruption of the successor’s tooth (translation or transmigration), associated pathologies (such as cysts, tumors, and odontoma under the primary tooth), the presence of supernumerary teeth in the surrounding area, a primary tooth with ankyloses/infra-occlusion, untreated dental caries (dental cavities) with or without pulp involvement, endodontic treatment (pulpotomy or pulpectomy), and dental fillings/dental restorations.

**Figure 1:**
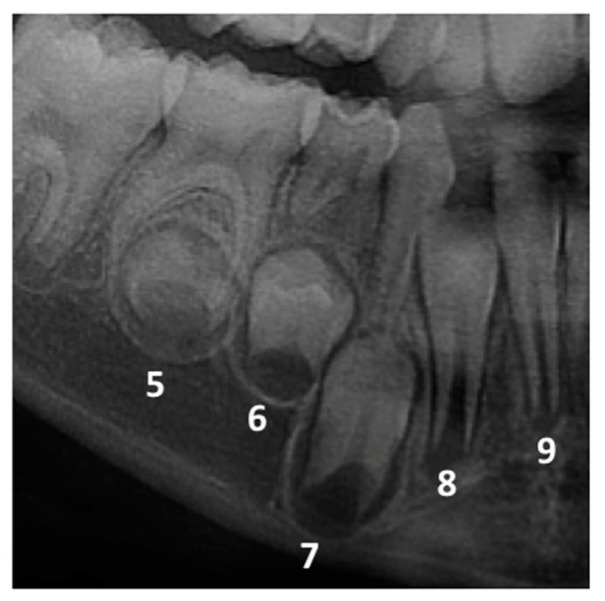
Nolla’s developmental stages 5, 6, 7, 8 and 9 in permanent premolars. Stage 5—crown almost completed; Stage 6—crown completed. Stage 7—1/3 root completed; Stage 8—2/3 root completed; and Stage 9—Root completed with the apex open.

**Figure 2:**
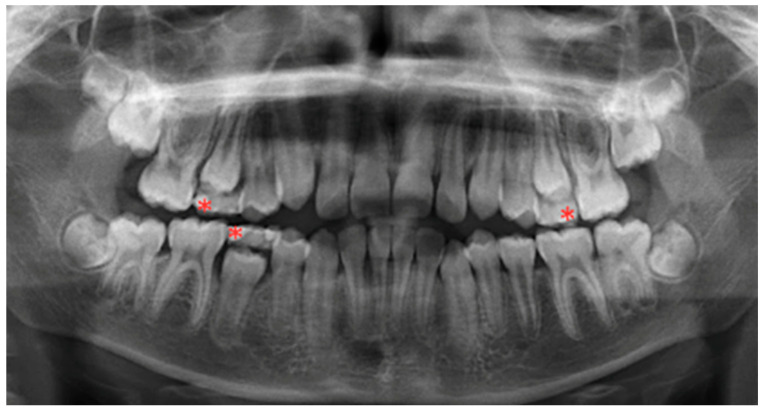
Orthopantomograph of a child in mixed dentition. * demonstrated primary teeth with the successor premolar in the Nolla’s stage 8; however, no alveolar crypt was observed, and teeth were not considered PPT.

The dental development of the permanent successors of each included primary tooth was assessed according to Nolla’s stage. The technique introduced by Nolla assigns each tooth a certain stage from 0 (absence of crypt) to 10 (apical end of the tooth root canal completed) [9]. Examples of Nolla’s stages 5, 6, 7, 8 and 9 are demonstrated in Figure 1. Nolla stage 8 is described as having two-thirds of the root completed [9,10], and the tooth has emerged into the oral cavity. Therefore, the primary tooth was considered retained when its successor permanent tooth was in Nolla stage 8 and below the alveolar crypt, Nolla stage 9 or Nolla, stage 10. The cases of Nolla stage 8 with the permanent tooth already reaching the alveolar crypt (alveolar crest) were not considered to be PPT, as demonstrated in Figure 2.

Therefore, only idiopathic cases of PPT associated with the delayed permanent tooth eruption of its successor’s tooth were considered.

### 2.3. Genomic DNA Extraction and Allelic Discrimination Analysis of COX2

A sample of buccal cells was collected from each of the included children for DNA analysis using sterile disposable cytobrushes. The samples were collected by swiping and rolling a cytobrush over the side (right and left), 2–3 times on the inner mucosa of the cheek and against the base of the tongue. Then, the samples were stored at −20 °C until processing for DNA extraction. The genomic DNA extraction followed an established protocol. Briefly, an extraction solution (Tris-HCl 10 mM, pH 7.8; EDTA 5 mM; SDS 0.5%) containing proteinase K (100 ng/mL) was added to cause the lysis of the cells and proteins from the saliva samples. The non-digested proteins were removed using ammonium acetate, and the DNA was precipitated with isopropanol. Later the DNA was resuspended in a TE buffer (10 mM Tris (pH 7.8) and 1 mM EDTA) and stored at −20 °C for future analysis. More details on DNA extraction and purification can be found in Küchler et al. [11]. The DNA’s concentration (estimated by measuring the absorbance at 260 nm) and purity (estimated by measuring the ratio of absorbance at 260 nm and 280 nm) were evaluated by spectrophotometry using NanoDrop 1000 (Thermo Scientific, DE, Waltham, MA, USA).

The potential SNPs in *COX2* (chromosome 1) were screened from the dbSNP database (http://www.ncbi.nlm.nih.gov/snp/ (4 April 2022)) and SNPinfo (http://snpinfo.niehs.nih.gov/ (4 April 2022)). The SNP selection was based on their MAF (minor allele frequency) of ≥10% in the global population and the possible clinical impact, taken from previous publications (http://www.ncbi.nlm.nih.gov/snp/ (4 April 2022)). Therefore, genotyping analyses were performed to investigate two SNPs in *COX2*: an upstream variant (rs689466; −1195 G/A) and an untranslated region (rs5275; +665 T/C). The characteristics of the selected SNPs are presented in Table 1. The allelic discrimination reactions were carried out using real-time polymerase chain reactions (PCR), TaqMan technology (Applied Biosystems^®^, StepOnePlus Real-Time PCR System, Thermo Fisher Scientific, Foster City, CA, USA). The real-time PCR reactions were performed in a total volume of 3 mL per well as follows: 4 ng DNA/reaction in 1.5 mL of sterile water, 1.5 mL of TaqMan^®^ Universal PCR Master Mix, and 0.075 of custom TaqMan^®^SNP genotyping assays (Applied Biosystems) specific to each SNP studied. The probes and master mix were obtained from Applied Biosystem (Foster City, CA, USA). A negative control without template DNA was used in each run. In addition, 20% of the sample was randomly selected for repeated analysis and demonstrated a concordance of 100%.

Thermal cycling was performed by starting with a holding cycle of 95° for 10 min, followed by 45 amplification cycles of 92° for 15 s and 60° for 1 min. The examiner in the molecular biology laboratory was blinded to the samples’ group during the genotyping analysis.

Patients whose DNA samples failed twice in the genotyping reaction were excluded from further analysis.

### 2.4. Statistical Analysis

For statistical analysis, a child was assigned to the PPT group when they had at least one tooth diagnosed as PPT. The Hardy–Weinberg Equilibrium (HWE) was assessed for each studied SNP by a chi-square test (wpcalc.com/en/equilibrium-hardy-weinberg (22 May 2022). A chi-square test was also used to compare the allele and genotype distributions (in the co-dominant model and recessive model) among the control and PPT groups using the software Epi Info 7.2. Haplotype analysis was also performed. The odds ratio (OR) and 95% Confidence Intervals (CI) were calculated. A T-test was used for the comparison of the means and standard deviations (SD) among the groups. The significance level was established as *p* < 0.05 (alpha of 5%) for all comparisons.

## 3. Results

**Figure 3:**
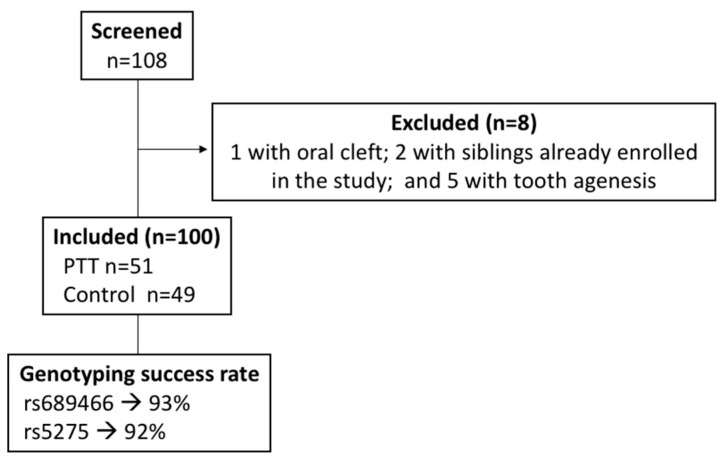
Flow diagram.

Among the 108 patients initially screened, one patient with cleft lip and palate and two patients that had brothers already enrolled in the study were excluded in the anamnesis stage. Five patients had tooth agenesis diagnosed in the radiographic analysis and were also excluded. Therefore, a total of 100 children were finally included in the study. Fifty-one children had at least one PPT and were allocated to the PPT group, while 49 children did not present with PPT and were classified as controls (Figure 3).

The characteristics of the PPT and control groups and the comparisons among them are presented in Table 2. The number of primary teeth presented in the oral cavity ranged from 0 to 13 (mean of 4.3; SD = 4.7) in the control group and from 1 to 12 (mean of 7.0; SD = 3.8) in the PPT group, in which there was a statistically significant difference between the groups (*p* = 0.002). Age and gender were not statistically significant among the different control and PPT groups (*p* = 0.446 and *p* = 0.548, respectively).

In the PPT group, the number of teeth persistent in the oral cavity ranged from one to eight. The mean was 1.56 (SD = 2.01).

Both studied SNPs were consistent with the Hardy-Weinberg Equilibrium: HWE ^chi-square^ = 2.07 for the rs5275 and HWE ^chi-square^ = 0.666 for rs689466.

The genotype and allele distributions according to the PPT group and control group are presented in Table 3. The genotype distribution was associated with PPT in the co-dominant model comparing TC versus TT (*p* = 0.006; OR = 0.28, CI 95% = 0.11–0.71) for the SNP rs5275. The individuals that carry two T alleles (TT) compared with individuals that carry at least one C allele (C + TC) had an almost three times higher chance of presenting with PPT (*p* = 0.012; OR = 2.99, CI 95% = 1.28 to 6.95–recessive model) also for the SNP rs5275.

For the SNP rs689466, a statistically significant difference was not observed in any of the models: allele distribution genotype distribution in the co-dominant and in the recessive models (*p* > 0.05).

Haplotypes of the two SNPs were analyzed and are presented in Table 4. The haplotype C-A for the SNPs rs5275 and rs689466, respectively, was more frequent in the control group and was significantly associated with PPT (*p* = 0.042).

## 4. Discussion

The eruption of both primary and permanent teeth is a physiological process during childhood; however, the time at which a primary tooth exfoliates and a tooth erupts into the oral cavity varies according to each child [3]. Although some systemic and local factors have been associated with PPT, the fact that the majority of the cases are idiopathic [4] suggests that genetic factors can influence the presence of PPT. In fact, in recent decades, some studies have attempted to investigate different conditions associated with tooth eruption disturbances. The genetic etiology behind a primary failure of eruption, which is a rare disorder defined as incomplete tooth eruption despite the presence of a clear eruption pathway, has been explored by some research groups. A systematic review pooled and critically revised some studies and their results. The authors identified that a total of 51 different variations of the *PTH1R* (parathyroid hormone 1 receptor) gene were associated with the primary failure of eruption [12]. More recently, some studies have also raised the suggestion that the mild forms of tooth eruption alterations, such as delayed tooth eruption with or without PPT, also have a genetic background involved in its etiology [4,13,14,15]. Variations (SNPs) in genes such as the receptor activator of NF-κB ligand (RANKL) [13] and Matrix metalloproteases 8 (MMP8) [14] could be involved in this common condition. Since all cases of tooth agenesis (excluding third molars agenesis) were excluded from our study, it is worth mentioning that all PPT cases also present with the delayed permanent tooth eruption of its successor. Therefore, in the present study, we investigated if common SNPs in *COX2* are involved in PPT associated with delayed permanent tooth eruption risk.

SNPs can be used as genetic markers to detect an association between a gene and a disease [7]. In our study, we were searching for possible candidate genes and SNPs for PPT, as the understanding of the genetic mechanisms involved in tooth eruption is important for dentists, especially orthodontists and pediatric dentists. In our study, we decided then to investigate a novel gene once we hypothesized that *COX2* could be involved in a higher risk of PPT due to its role in the resorption process. Primary root resorption and bone resorption and remodeling are required for the eruption of the majority of permanent teeth. Root resorption has a regulation similar to bone resorption, and both processes share the same molecular background. The main biological difference between root and bone is that bone undergoes constant physiological renovation, whereas, in teeth, the only resorption that is physiological is the one that occurs in primary teeth [16,17]. Some studies investigated the mechanism of root resorption and found that the process is similar to bone resorption [16,18]. Additionally, odontoclasts, cementoclasts, and osteoclasts share similar characteristics, a similar gene expression profile, and mineralized–tissue-resorbing activity [16]. *COX2* plays a well-known role in bone resorption. Some studies showed that during orthodontic tooth movement, *COX2* is expressed and plays an important role. *COX2* is involved in tooth movement during orthodontic treatment through alveolar bone remodeling resorption processes [19,20]. Both clinical and in vitro experiments showed that *COX2* is expressed by human periodontal ligament cells [20,21]. The periodontal ligament cells of primary teeth also express COX2. A study comparing the periodontal ligament of human primary and permanent teeth observed that primary teeth had higher *COX2* expression and a lower expression of OPG. The authors concluded that the more intense immunoreaction to COX2 and the occurrence of clastic cells and active macrophages might indicate that proinflammatory cytokines are present in the periodontal ligament, stimulating a down-regulation of OPG (Osteoprotegerin) and an up-regulation of RANKL, and activating resorption of tooth tissues [6]. RANKL signaling regulates osteoclast formation, activation, and survival in normal bone modeling and remodeling, while OPG protects bone from excessive resorption by binding to RANKL and preventing it from binding to RANK (receptor activator of NF-κB) [22].

Although the *COX2* gene is highly polymorphic and many SNPs have been identified until now (www.ncbi.nlm.nih.gov/snp/) (1 July 2022), only a few SNPs have been associated with clinical conditions so far. In our study, we found that the SNP rs5275 in COX2 and the haplotype C-A in rs5275-rs689466 could be involved in a higher risk to present PPT. The rs5275 is an SNP located within the 3′ UTR region (untranslated region). The post-transcriptional regulation of COX2 is dependent, in part, on sequences within the 3′ UTR of the COX2 mRNA (messenger RNA). Therefore, the rs5275 is supposed to affect mRNA stability and/or translation efficiency [23]. A meta-analysis performed pooling studies that investigated the SNP rs5275 and periodontitis, and an association of this SNP with periodontal disease was observed [24]. Although periodontal disease and PPT are different conditions, the results observed in the meta-analysis support the role of rs5275 SNP in alveolar bone/tooth resorption. Bioinformatics showed that the SNP rs689466 creates a c-MYB binding site, which results in higher transcriptional activity of the *COX2* gene [25]. In our study, this SNP was associated with PPT only in the haplotype analysis. These results suggest that it is the SNP rs5275 that influences *COX2* promoter activity in the context of PPT.

Our study has some weaknesses and strengths. This is one of the first studies to explore genes involved in PPT. However, it is important to highlight that our study is not a population-based study large enough to have sufficient power to detect small associations. Furthermore, because only a few SNPs in the *COX2* gene have a minor allele frequency higher than 20%, we were not able to select SNPs that represent the majority of the sequence variations of the gene. We emphasize that our findings must be replicated in independent study groups.

Briefly, our results support the hypothesis that common SNPs in the *COX2* signaling pathway may act as potential predictors of PPT risk associated with delayed permanent tooth eruption. With our growing knowledge of SNPs and their involvement in different conditions, we will be able to identify children at a higher risk for oral conditions.

## 5. Conclusions

Our study demonstrated that a single nucleotide polymorphism rs5275 and a haplotype in the gene encoding *COX2* are associated with persistent primary tooth and delayed eruption of the permanent successor tooth.

## Figures and Tables

**Table 1:** Description of the studied SNPs in *COX2* gene.

SNP	Also Known	Function	Global Minor Allele Frequency	Hardy-Weinberg χ2
rs689466	−1195 G/A	Upstream Variant	0.217	1.36
rs5275	+665 T/C	3′ UTR region	0.400	2.07

**Table 2:** Characteristics and comparisons of groups.

Variables	Control (n = 49)	PPT (n = 51)	*p*-Value
Age in months Mean (SD)	133.1 (SD 19.4)	130.3 (SD 16.9)	0.446 *
Number of primary teeth Mean (SD)	4.3 (SD 4.7)	7.0 (SD 3.8)	0.002 *
Gender *n* (%)			
Male	26 (53.1%)	24 (47.1%)	0.548 ^#^
Female	23 (46.9%)	27 (52.9%)	

Note: * *t*-test was used. ^#^ Chi-square was used. Bold form indicates a statistically significant difference (*p* < 0.05).

**Table 3:** Genotype and allele distribution according to PPT and controls.

SNP	Genotype	Control *n* (%)	PPT *n* (%)	*p*-Value	OR (CI 95%)
rs689466 (−1195 G/A)	AA	26 (57.8)	26 (54.1)	Reference	
	GA	17 (37.8)	20 (41.7)	0.706	1.17 (0.51–2.76)
	GG	2 (4.4)	2 (4.2)	>0.999	1.00 (0.14–6.75)
	A	69 (76.6)	72 (75.0)	Reference	
	G	21 (23.3)	24 (25.0)	0.790	1.09 (0.57–2.13)
rs5275 (+665 T/C)	TT	12 (26.7)	25 (52.1)	Reference	
	TC	30 (66.7)	18 (37.5)	**0.006**	0.28 (0.11–0.71)
	CC	3 (6.6)	5 (10.4)	0.782	1.25 (0.29–6.31)
	T	54 (60.0)	68 (70.8)	Reference	
	C	36 (40.0)	28 (29.2)	0.098	0.67 (0.36–1.23)

Note: OR means odds ratio; CI means confidence interval. Bold form indicates a statistically significant difference (*p* < 0.05).

**Table 4:** Haplotype association analysis between controls and PPT groups.

Haplotype	Frequency		*p*-Value
rs5275-rs689466	Control	PPT	
T-G	0.387	0.392	0.836
T-A	0.816	0.784	0.885
C-A	0.653	0.450	**0.042**
C-G	0.245	0.156	0.271

Note: Bold form indicates a statistically significant difference (*p* < 0.05).

## Data Availability

Data are available from the corresponding author upon reasonable request.
